# Atherosclerotic Plaque Crystals Induce Endothelial Dysfunction

**DOI:** 10.3390/ijms26199758

**Published:** 2025-10-07

**Authors:** Jishamol Thazhathveettil, Sherin Aloysius Gomez, Deborah Olaoseeji, Rongrong Wu, Allan Sirsjö, Geena Varghese Paramel

**Affiliations:** 1 Department of Clinical Research Laboratory, Faculty of Medicine and Health, Örebro University, 701 82 Örebro, Sweden; jishamol.thazhath-veettil@regionorebrolan.se; 2 Department of Biosciences, University of Skövde, 541 28 Skövde, Sweden; sherinaloysius@gmail.com; 3 School of Medical Sciences, Örebro University, Campus USÖ, 701 82 Örebro, Sweden; debbiefunmy@gmail.com (D.O.); rongrong.wu@oru.se (R.W.); allan.sirsjo@oru.se (A.S.)

**Keywords:** cholesterol crystal (CC), monosodium urate (MSU), neutrophil extracellular traps (NETs), atherosclerosis, endothelial dysfunction

## Abstract

Endothelial dysfunction is an early driver of atherosclerosis, yet the direct impact of endogenous crystals such as cholesterol crystals and monosodium urate on endothelial activation remains incompletely understood. In this study, we examine how crystalline stimuli modulate human umbilical vein endothelial cells by assessing inflammatory signaling, mitochondrial respiration, and neutrophil recruitment. Using dose- and time-controlled experiments, we show that CC and MSU are internalized by endothelial cells, activating NF-κB and STAT3 signaling pathways and inducing a robust pro-inflammatory cytokine profile. Notably, CC caused marked mitochondrial dysfunction, evidenced by impaired respiratory capacity and loss of membrane potential, revealing a novel bioenergetic vulnerability in endothelial cells. Both direct crystal stimulation and exposure to crystal-primed conditioned media triggered endothelial adhesion molecule expression and promoted neutrophil adhesion, indicating that soluble mediators released upon crystal stimulation can propagate vascular inflammation. These findings demonstrate that crystalline stimuli are potent vascular danger signals capable of driving endothelial inflammation, mitochondrial impairment, and immune cell engagement, which are hallmarks of early atherogenesis. By elucidating these multifaceted endothelial responses, this study provides important mechanistic insights into how crystal-induced signals may contribute to vascular dysfunction and the early stages of atherogenesis.

## 1. Introduction

The endothelium, the innermost cell layer of blood vessels, plays a crucial role in maintaining vascular homeostasis by regulating vascular tone, preventing platelet and leukocyte adhesion, and controlling vascular proliferation [1]. However, when endothelial function is compromised, it leads to endothelial dysfunction, which is characterized by increased permeability, elevated cytokine production, and enhanced adhesion of platelets and leukocytes to the vessel wall. This dysfunction is implicated in the development and progression of various vascular diseases, including atherosclerosis and hypertension. Endothelial dysfunction is considered to be a critical early event in atherosclerosis, as it promotes the formation of plaque through mechanisms that enhance inflammation and vascular injury [2]. Key to this process are adhesion molecules such as ICAM1 and VCAM1, which facilitate the adhesion and migration of leukocytes into the vessel walls, triggering inflammatory responses [3]. The activation of these inflammatory pathways is driven by activation of transcription factors like NF-κB, which further amplifies inflammatory response [4]. This vicious cycle of oxidative stress and inflammation not only accelerates endothelial damage, but also contributes to the pathogenesis of atherosclerosis and other vascular disorders [5].

Crystalline materials such as cholesterol crystals (CC) and monosodium urate crystals (MSU) have emerged as active contributors to sterile inflammation within the vascular system. CC are frequently observed within atherosclerotic plaques and are known to initiate inflammatory signaling, disrupt cellular membranes, and promote the release of interleukin-1β through NLRP3 inflammasome activation [6]. In addition to these inflammatory effects, CCs disrupt endothelial integrity, and enhance monocyte adhesion, thereby accelerating lesion progression and vascular dysfunction [7,8]. Furthermore, CC formation contributes to smooth muscle cell death during plaque evolution, linked to impaired autophagy and metabolic stress [9]. Overall, CCs are central to the progression of atherosclerosis by promoting plaque instability, inflammation, and endothelial dysfunction, underscoring role of CC as critical targets in cardiovascular research.

Similarly, MSU, traditionally associated with gout, has been identified within coronary arteries and atherosclerotic lesions. Histopathological and imaging studies reveal MSU accumulation in inflamed plaque regions, especially in areas rich in macrophages and neovessels [10]. Functionally, MSU crystals are potent activators of NLRP3 inflammasomes, driving IL-1β secretion and endothelial activation. Recent experimental studies also show that MSU induces MAPK and p38 signaling in immune cells to promote inflammation and adhesion molecule expression [11,12,13]. Epidemiological evidence further connects elevated uric acid with endothelial dysfunction and increased cardiovascular risk [14]. These findings highlight the potential relevance of MSU in vascular inflammation and atherogenesis.

Despite accumulating evidence linking these crystals to vascular inflammation, their direct cellular effects on endothelial physiology remain insufficiently characterized. It is unclear whether CC and MSU modulate distinct or overlapping pathways of endothelial activation, barrier disruption, inflammatory mediators, and mitochondrial impairment. This study aims to fill that gap by systematically investigating the impact of CC and MSU on endothelial function and immune cell interactions.

In this study, we explore how cholesterol and monosodium urate crystals influence endothelial cells, focusing on their ability to activate inflammatory signaling, modulate adhesion molecule expression, and affect mitochondrial function, thereby providing insight into their potential role in early atherogenesis.

## 2. Results

### 2.1. Uptake of Cholesterol Crystals and Monosodium Urate Crystals by HUVECs

**Figure 1:**
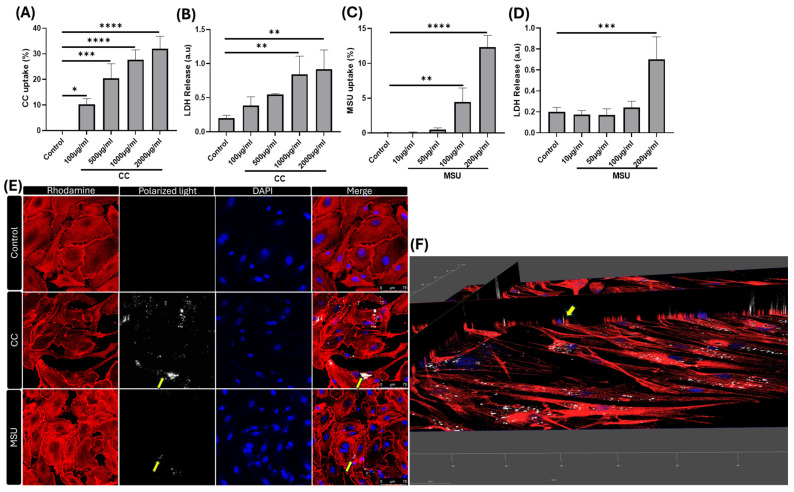
Uptake of CC and MSU in HUVECs. Dose-dependent uptake and LDH release in response to CC (**A**,**B**) and MSU (**C**,**D**) in HUVECs for 24 h. Confocal microscopy was performed to visualize CC uptake in HUVECs at 24 h (CC is shown in white by polarized light (yellow arrows), actin in red by Rhodamine phalloidin, and nucleus in blue by DAPI staining). Scale bar: 75 μm (**E**). Z-stacking 3D image showing CC, nucleus, and actin filament (yellow arrows) in the same plane, confirming the uptake. Scale bar: 50 μm, objective 20× (**F**). Data represents the mean ± SD of three independent experiments in triplicate. * *p* < 0.05, ** *p* < 0.01, *** *p* < 0.001, **** *p* < 0.0001.

Building on our previous work, which demonstrated that primary vascular smooth muscle cells (VSMCs) can internalize CC, we next examined whether HUVECs also respond to crystalline stimuli. Notably, previous studies have shown that cholesterol crystals may nucleate within endothelial cells and subsequently be secreted into subendothelial space, where they continue to grow as more lipids and crystalline material accumulate [15]. We observed a clear dose-dependent uptake of both CC and MSU crystals by HUVECs (Figure 1A,C). At a CC concentration of 0.5 mg/mL, approximately 20% of HUVECs exhibited saturated intracellular accumulation of cholesterol crystals. This uptake was associated with a moderate dose-dependent increase in cytotoxicity from 1 mg/mL onward (Figure 1E,F), whereas MSU exposure resulted in detectable cytotoxicity at 200 µg/mL. The process may contribute to a self-perpetuating cycle of endothelial dysfunction and crystal uptake, particularly in regions of lipid-rich atherosclerotic lesions.

To explore the relevance of MSU crystals in the vascular context, we considered the pathological localization of MSU. Previous studies have demonstrated the presence of MSU deposits co-localized with CD31-positive micro-vessels and CD68-positive macrophages in the shoulder regions of atherosclerotic plaques areas known to harbor active inflammation and neovascularization [16]. HUVECs have displayed dose-dependent uptake of MSU crystals in vitro. At a concentration of 0.1 mg/mL, around 5% of the cells internalized MSU (Figure 1C). At higher concentrations, MSU exposure led to a marked increase in cytotoxicity, indicating that, like CC, MSU crystals can directly impair endothelial viability in a concentration-dependent manner (Figure 1D). The Z-stack images from confocal microscopy reveal colocalization of nuclei (DAPI), F-actin (Rhodamine phalloidin), and cholesterol crystals (visualized by polarized light) within the same focal plane (Figure 1E,F), confirming the intracellular localization of CC in HUVECs.

Together, these findings demonstrate that HUVECs can internalize both CC and MSU crystals in a dose-responsive manner. Importantly, our in vitro observations align with in vivo evidence of MSU accumulation in inflamed, vascularized regions of human plaques, highlighting the potential role of crystal–endothelial interactions in vascular pathology.

### 2.2. Cholesterol Crystals and Monosodium Urate Crystals Induce Release of Inflammatory Cytokine in HUVECs

**Figure 2:**
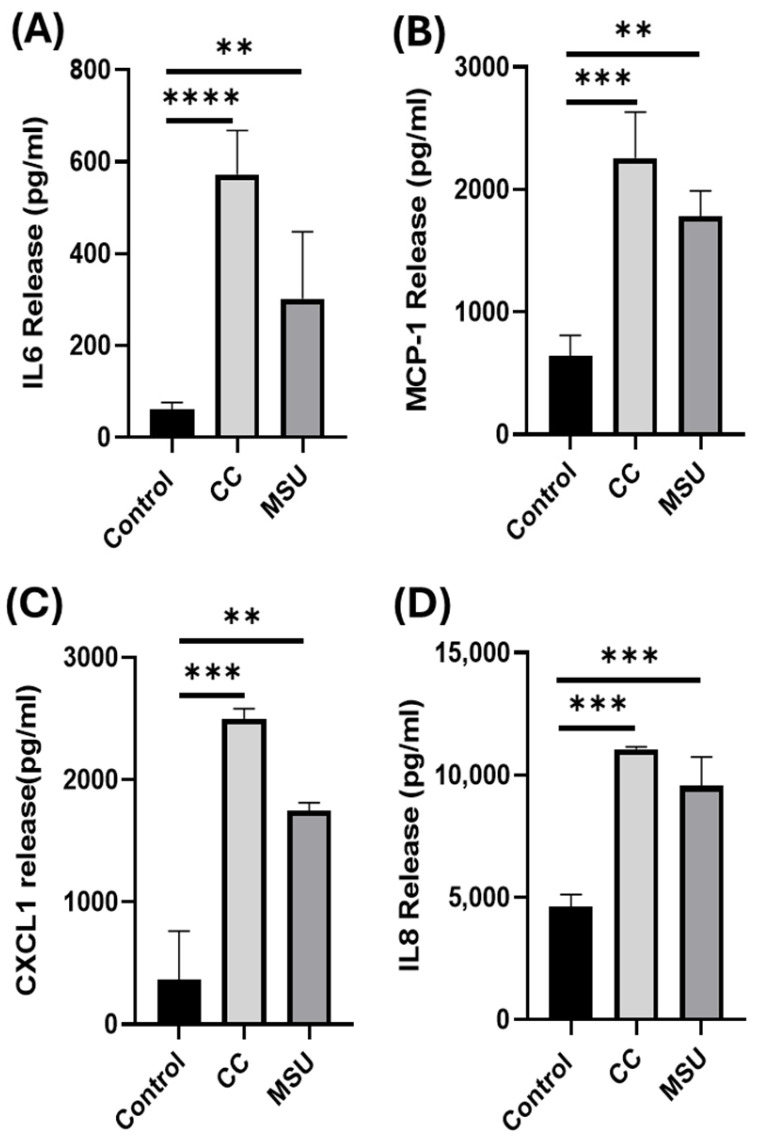
Cytokine release in response to CC and MSU in HUVECs. HUVECs were treated with CC and MSU for 24 h, and levels of IL-6 (**A**), MCP-1 (**B**), CXCL1 (**C**), and IL-8 (**D**) were measured in the cell culture supernatant. Data represents the mean ± SD of three experiments in triplicate. ** *p* < 0.01, *** *p* < 0.001, **** *p* < 0.0001.

To investigate the inflammatory response elicited by CC and MSU in HUVECs, we assessed the secretion of key pro-inflammatory cytokines and chemokines implicated in atherogenesis, including IL-6, MCP-1, CXCL1, and IL-8. Stimulation with both CC and MSU resulted in a marked increase in the release of inflammatory mediators, IL-6, MCP-1, CXCL1, and IL-8 under non-cytotoxic conditions (Figure 2). These findings indicate that crystalline stimuli can activate endothelial inflammatory pathways and promote the release of pro-atherogenic cytokines and chemokines in the absence of overt cytotoxicity.

### 2.3. Cholesterol Crystals and Monosodium Urate Crystals Induce Inflammatory Signaling Pathways in HUVECs

**Figure 3:**
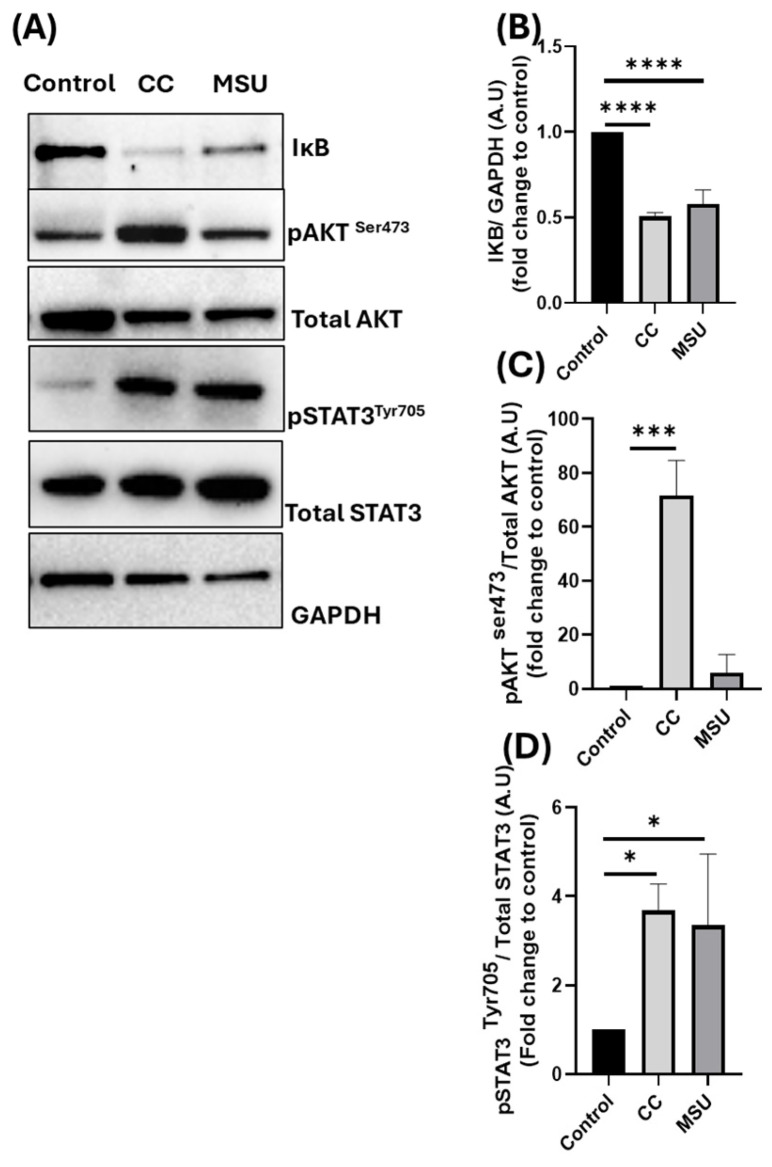
Signaling pathways induced in response to CC and MSU in HUVECs. HUVECs were treated with CC and MSU for 4 h, and the expression of key signaling proteins was measured using Western blot (**A**). The densitometry analysis of significantly altered proteins is shown for IκB (**B**), pAKT (**C**), and pSTAT3 (**D**). Data represents the mean ± SD of three independent experiments. * *p* < 0.05, *** *p* < 0.001, **** *p* < 0.0001.

To further explore the molecular signaling events underlying endothelial responses to crystalline particles, we investigated the activation of key pro-inflammatory and pro-survival pathways in HUVECs following exposure to CC and MSU. At non-cytotoxic concentrations, both CC and MSU induced phosphorylation of STAT3 (pSTAT3) (Figure 3A,D), a transcription factor associated with inflammatory signaling and endothelial activation. Additionally, a marked reduction in IκBα levels (Figure 3A,B) was observed with both stimuli, indicating activation of the NF-κB pathway, a hallmark of pro-inflammatory responses in endothelial cells. However, only CC-induced phosphorylation of AKT (Figure 3A,C), also a key regulator of inflammatory signaling, implicated the activation of the PI3K/AKT pathway. This differential signaling response suggests that CC elicits a broader and more potent activation profile in endothelial cells, engaging multiple inflammatory signaling pathways, including NF-κB, STAT3, and the AKT axis. These results provide mechanistic insights into how endothelial cells sense and respond to distinct crystal types. While both CC and MSU trigger inflammatory activation, the selective activation of AKT by CC may reflect a distinct upstream receptor engagement or differential intracellular sensing that amplifies pro-inflammatory signaling in endothelial cells.

### 2.4. Cholesterol Crystals Induce Endothelial Activation and Neutrophil Adhesion

**Figure 4:**
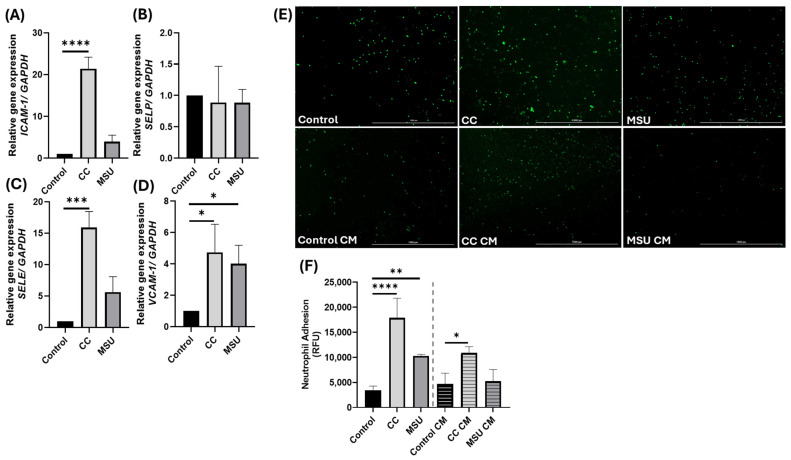
Neutrophil adhesion induced in response to CC in HUVECs. HUVECs were treated with CC and MSU for 24 h, and the expression of key adhesion genes were measured using qPCR (**A**–**D**). Representative image showing Calcein AM-labeled neutrophil adhesion on endothelial cells in response to CC, MSU, and conditioned medium (CM) from HUVECs treated with and without CC and MSU for 24 h. Scale bar: 1000 μm, objective 4× (**E**). Quantification of neutrophil adhesion on endothelial cells in response to treatment. Dotted line indicates separation of directly treated groups and CM groups (**F**). Data represents the mean ± SD of three independent experiments in triplicate. * *p* < 0.05, ** *p* < 0.01, *** *p* < 0.001, **** *p* < 0.0001.

To elucidate the pro-inflammatory impact of crystalline particles on the endothelium, we assessed adhesion molecule gene expression and neutrophil recruitment in HUVECs following exposure to CC and MSU crystals. Quantitative gene expression analysis revealed that CC stimulation at non-cytotoxic concentrations markedly induced the transcription of *ICAM1*, *VCAM1*, and *SELE* (encoding E-selectin), consistent with a classical endothelial activation phenotype (Figure 4A,C,D). In contrast, MSU exposure selectively upregulated *VCAM1* mRNA, without appreciable changes in *ICAM1* or *SELE* expression. Notably, expression of *SELP* (P-selectin) remained unchanged in response to crystals (Figure 4B).

Functionally, these transcriptional changes were reflected in a differential capacity to recruit neutrophils. CC-treated HUVECs exhibited a pronounced increase in neutrophil adhesion (Figure 4E,F), in line with the broad upregulation of surface adhesion molecules. MSU exposure also promoted neutrophil attachment (Figure 4E,F), albeit to a significantly lesser extent, which likely reflects its limited induction of the adhesion molecule repertoire.

To assess the role of soluble inflammatory mediators in amplifying endothelial activation, we treated HUVEC monolayers with a conditioned medium (CM) derived from CC- or MSU-stimulated endothelial cells. CM from CC-exposed HUVECs induced significant neutrophil adhesion on previously unstimulated endothelial cells, implicating a paracrine mechanism of inflammation propagation (Figure 4E,F). In contrast, CM from MSU treated HUVECs failed to elicit a similar response, further highlighting the restricted inflammatory profile induced by MSU crystals (Figure 4E,F).

Collectively, these findings demonstrate that CCs elicit a robust endothelial pro-inflammatory response involving both direct transcriptional activation of adhesion molecules and the secretion of soluble factors capable of priming adjacent endothelial cells for neutrophil recruitment. MSU crystals, while capable of inducing limited activation, do not trigger paracrine inflammatory amplification, emphasizing the qualitative differences in the endothelial responses to structurally distinct crystalline stimuli.

### 2.5. Cholesterol Crystals Disrupt Endothelial Barrier Function and Selectively Induce Net Formation

**Figure 5:**
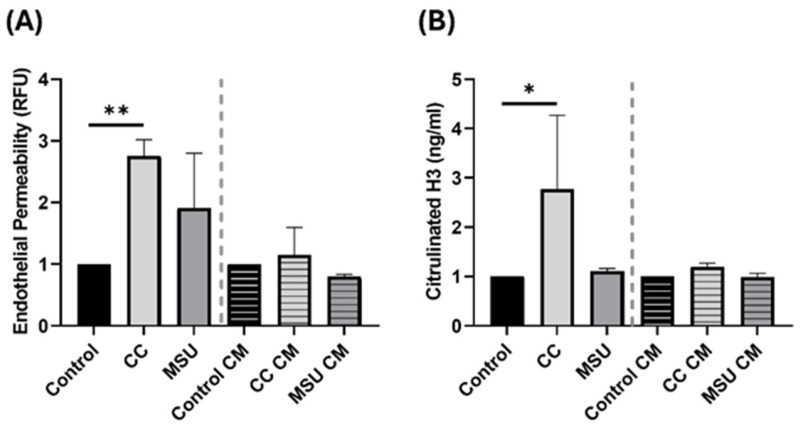
Vascular permeability and NETs formation induced in response to CC in HUVECs. HUVECs were treated with CC, MSU, and conditioned medium (CM) from HUVECs, and were treated with and without CC and MSU for 24 h. Endothelial permeability (**A**) and NETs formation (**B**) were measured. Dotted line indicates separation of directly treated groups and CM groups. Data represents the mean ± SD of three independent experiments in triplicate. * *p* < 0.05, ** *p* < 0.01.

To evaluate the impact of crystalline stimuli on endothelial barrier integrity, trans endothelial permeability was assessed in HUVEC monolayers following exposure to CC, MSU, and CM collected from CC- or MSU-stimulated endothelial cells. Direct exposure to CC resulted in a significant increase in endothelial permeability (Figure 5A), indicating disruption of the endothelial barrier. In contrast, MSU and CM from crystal-stimulated HUVECs had no effect on barrier function, suggesting that direct contact with cholesterol crystals is required to compromise endothelial integrity (Figure 5A). To assess the capacity of crystalline particles to activate neutrophils, NET formation was quantified following stimulation with CC, MSU, or CM derived from crystal-exposed HUVECs. CC induced a robust NET response, as determined by elevated levels of citrullinated histone H3 (Figure 5B). In contrast, MSU and all CM conditions failed to elicit NET release, indicating that NETosis is specifically triggered by direct neutrophil exposure to cholesterol crystals, and not by endothelial derived soluble mediators (Figure 5B).

Together, these findings highlight the ability of CC to simultaneously impair endothelial barrier function and directly induce neutrophil extracellular trap formation, two critical processes implicated in vascular inflammation and thrombosis. MSU lacks these effector functions under equivalent conditions, underscoring distinct immunovascular activation profiles between the two crystal types.

### 2.6. Cholesterol Crystals Impair Mitochondrial Respiration in HUVECs

**Figure 6:**
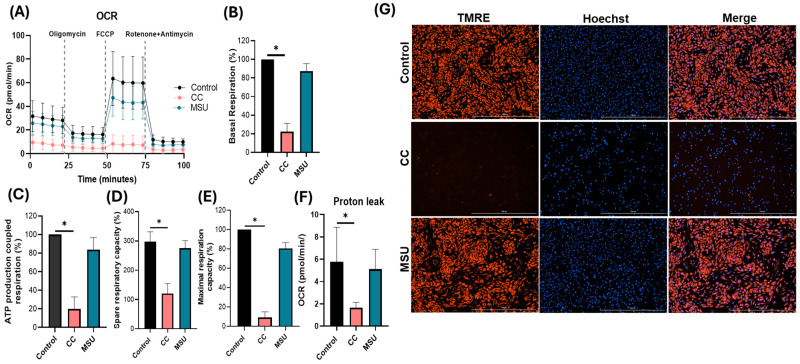
Cholesterol crystals impair mitochondrial respiration in HUVECs. Representative traces of oxygen consumption rate (OCR) of HUVECs following sequential addition of oligomycin, FCCP, and rotenone/antimycin are shown on the left (**A**). Corresponding bar graph quantification of key respiratory parameters derived from the raw OCR data. Quantification of basal respiration (**B**), ATP production-coupled respiration (**C**), spare respiratory capacity (**D**), maximal respiration capacity (**E**), and proton leak (**F**). Mitochondrial membrane potential in response to CC and MSU was assessed using TMRE fluorescence (red); nuclei were counterstained with Hoechst 33342 (blue). Images were acquired at 4× objective. Scale bar: 1000 µm (**G**). Data represents the mean ± SD of three independent experiments in duplicates. * *p* < 0.05.

Mitochondrial function in HUVECs was evaluated using an extracellular flux analyzer following exposure to CC or MSU at non-cytotoxic concentrations. CC markedly suppressed basal oxygen consumption, indicating reduced steady-state respiratory activity (Figure 6A,B). Oligomycin-sensitive (ATP-linked) respiration was significantly decreased, consistent with impaired mitochondrial ATP production and diminished coupled respiration (Figure 6C). Furthermore, CC treated cells exhibited reduced spare respiratory capacity and lower maximal respiration following FCCP uncoupling, demonstrating a loss of metabolic flexibility under stress conditions (Figure 6D,E). A concurrent decrease in proton leak associated respiration was observed, suggesting constrained electron transport chain dynamics and/or altered inner mitochondrial membrane properties under CC exposure (Figure 6F). By contrast, MSU treatment did not significantly alter basal, ATP-linked, maximal, spare, or proton leak respiration relative to control (Figure 6A–F), indicating that MSU does not measurably compromise mitochondrial bioenergetics in endothelial cells under the tested conditions. To complement functional flux metrics, mitochondrial membrane potential was assessed using TMRE staining (Figure 6G). CC exposure resulted in reduced TMRE fluorescence intensity, consistent with partial depolarization of the mitochondrial inner membrane (Figure 6G). TMRE signals remain unchanged in MSU treated cells, further supporting a lack of mitochondrial dysfunction in response to MSU (Figure 6G).

Collectively, these data identify cholesterol crystals as a potent disruptor of endothelial mitochondrial function, compromising both energetic output and reserve capacity while reducing membrane potential. MSU crystals appear bioenergetically inert in comparison, reinforcing differential pathogenic potential between these crystalline stimuli.

## 3. Discussion

This study provides a comprehensive analysis of the effects of CC and MSU on HUVECs, focusing on the capacity of crystals to modulate the key aspects of endothelial physiology, including particle uptake, inflammatory signaling, adhesion molecule expression, barrier integrity, and mitochondrial function. Our findings reveal that both CC and MSU can be internalized by endothelial cells and initiate inflammatory responses, yet they engage distinct sets of downstream processes, leading to varied cellular responses that depend on factors such as crystal concentration, duration of exposure, receptor availability, and the broader inflammatory microenvironment.

The ability of HUVECs to internalize crystalline particles highlights their active role in innate immune sensing within the vascular compartment. Using flow cytometry and confocal microscopy, we demonstrate a dose-dependent uptake of both CC and MSU. Supporting the translational relevance of our findings, recent clinical imaging with dual-energy computed tomography (DECT) has confirmed the presence of MSU crystal deposits in coronary arteries, including the vascular wall, underscoring the translational relevance of direct crystal–endothelium interactions in coronary artery disease [16]. Prior studies also show that endothelial cells internalize native LDL and initiate intracellular cholesterol crystallization beneath the endothelium, implicating them in early crystal formation and vascular inflammation [15]. Importantly, the extent of uptake and intracellular accumulation varied with concentration, and this uptake was associated with mild cytotoxicity, suggesting a threshold beyond which crystal burden may become pathogenic.

Building on our uptake findings, CC and MSU triggered the secretion of pro-inflammatory cytokines and chemokines, including IL-6, MCP-1, CXCL1, and IL-8, under non-cytotoxic conditions. While cholesterol crystals have been linked to endothelial cytokine release through inflammasome and complement-dependent mechanisms [17,18], and MSU can trigger IL-1 and TNF-α release from monocytes [19], our results extend these observations by showing that both crystals directly activate endothelial inflammatory programs without monocyte intermediates.

To gain insight into the molecular pathways underlying this cytokine release, we next investigated the intracellular signaling responses initiated by CC and MSU in HUVECs. Three key pathways were examined: the NF-κB pathway, the STAT3 signaling axis, and the PI3K/AKT pathway. Both CC and MSU induced IκBα degradation, consistent with NF-κB activation and downstream adhesion/cytokine transcription; this aligns with prior reports of CC-driven NF-κB-dependent ICAM1/VCAM1 induction and monocyte adhesion [8] and MSU-triggered NF-κB in myeloid models (attenuated by curcumin), which could be attenuated by curcumin, a known NF-κB pathway inhibitor [20]. Additionally, we observed phosphorylation of STAT3 following exposure to both crystals. Our observation of STAT3 phosphorylation following MSU exposure in endothelial cells aligns with broader evidence indicating that MSU can activate STAT3 in target tissues. For instance, in a diabetic nephropathy model, MSU-induced NLRP3 pathway activation coincided with increased p-STAT3 expression in renal fibroblasts [21]. This supports the broader concept that MSU engagement can trigger STAT3-mediated inflammatory and fibrotic signaling across different cell types. To our knowledge, STAT3 activation by CC has not been previously reported in endothelial cells, suggesting a novel role for this transcription factor in mediating crystal-induced inflammation.

Finally, CC selectively induced phosphorylation of AKT, implicating the activation of the PI3K/AKT pathway. Interestingly, prior studies have reported divergent effects of MSU on AKT signaling, with some showing induction and others reporting inhibition, depending on cell lineage, experimental parameters, and the broader inflammatory milieu [22,23]. In our model, MSU did not induce AKT phosphorylation in HUVECs under non-cytotoxic conditions. This lack of response suggests that AKT signaling may not be a predominant pathway in endothelial responses to MSU, or, alternatively, that its activation might require additional priming signals or pathological conditions not captured in our experimental design. The selective engagement of AKT by CC may amplify inflammatory signaling and enhance cytokine output, adhesion molecule expression, and leukocyte recruitment.

To assess downstream consequences of crystal-induced signaling, we examined adhesion molecule expression and neutrophil recruitment. CC induced a robust transcriptional upregulation of *ICAM1*, *VCAM1*, and *SELE*, consistent with classical endothelial activation, and produced strong neutrophil adhesion. MSU increased *VCAM1* expression without appreciable changes in *ICAM1* or *SELE*, yielding a weaker adhesion phenotype under our conditions. Notably, conditioned medium from CC-stimulated HUVECs was sufficient to promote neutrophil adhesion on naive monolayers, indicating the release of soluble mediators with paracrine activity, whereas conditioned medium from MSU lacked this effect, suggesting a more localized response. These results align with prior reports that CC upregulates *ICAM1* and *VCAM1* through NF-κB-dependent mechanisms [8,15] and that MSU can induce VCAM1 and E-selectin, promoting leukocyte–endothelial interactions [24,25]. However, the magnitude and kinetics of adhesion molecule expression may depend on the duration of crystal exposure. For instance, Chapman et al. reported that E-selectin expression in response to MSU peaks at 4 h and declines thereafter [24]. In our study, HUVECs were stimulated with MSU for 24 h, which may explain the absence of sustained E-selectin induction. These observations support the conclusion that MSU selectively increases VCAM1 expression during prolonged exposure, with limited effects on ICAM1 or SELE. Our findings are further aligned with a study by Liu et al. (2017), which demonstrated that MSU at 100 µg/mL induced VCAM1, but not ICAM1 expression in endothelial cells [25]. Notably, ICAM1 induction was only observed at higher MSU concentrations. This supports a concentration-dependent effect of MSU on adhesion molecule expression. Together, these data indicate that CC elicits broader adhesion programs and paracrine amplification, while MSU produces a more confined, VCAM1-dominant activation, underscoring the importance of exposure kinetics, concentration, and spatial context in endothelial inflammatory propagation.

Further, we evaluated the integrity of the endothelial barrier in response to crystal exposure. Direct treatment with CC increased trans endothelial permeability, indicating barrier disruption. This aligns with previous findings demonstrating that CC disrupts endothelial junctions by inducing tyrosine phosphorylation of VE-cadherin and α-catenin, thereby increasing endothelial permeability [7]. Neither MSU nor CM from crystal-treated cells altered permeability, suggesting that direct contact with CC is required to compromise endothelial integrity. These findings are relevant in the context of atherosclerotic plaque progression, where barrier disruption can facilitate leukocyte infiltration and lipid deposition.

NET formation was also assessed following exposure to crystals and CM. CC induced strong NET release, marked by elevated citrullinated histone H3. This is consistent with previous evidence, including our own prior findings, demonstrating that CC robustly triggers NET release [26,27]. Interestingly, we also observed that, while CC-CM from HUVECs failed to induce NET formation, our previous study showed that CM from CC-stimulated vascular smooth muscle cells triggered NET release [27]. This highlights a potentially cell type specific secretion of NET inducing factors and underscores the absence of such mediators in the secretome of CC-treated HUVECs. Furthermore, MSU did not elicit NET formation under the concentrations used in this study. However, it is important to note that previous studies have demonstrated NET induction by MSU at higher concentrations or under different priming conditions [28]. Thus, NET formation in response to MSU may be dose- and microenvironment-dependent, and the lack of response in our system should not be interpreted as an absence of potential NET-inducing capacity.

Lastly, we assessed the impact of crystalline exposure on endothelial mitochondrial function by evaluating both oxidative respiration and mitochondrial membrane potential. CC markedly impaired mitochondrial bioenergetics in HUVECs, as indicated by significant reductions in basal, ATP-coupled, maximal, and spare respiratory capacity, alongside a pronounced decline in mitochondrial membrane polarization. The observed bioenergetic deficits reflect compromised mitochondrial function, which may potentiate pro-inflammatory signaling and contribute to endothelial dysfunction. Although previous studies have described a cholesterol crystal-induced metabolic shift toward glycolysis in macrophages [29], the current study is the first to demonstrate mitochondrial vulnerability in human endothelial cells in response to CC under non-cytotoxic conditions. This finding suggests a previously unexplored mechanism by which cholesterol crystals disrupt vascular homeostasis via the direct impairment of endothelial metabolic resilience and mitochondrial integrity. In contrast, MSU crystals did not significantly perturb mitochondrial respiratory parameters or membrane potential, indicating preserved mitochondrial function in endothelial cells under the defined experimental conditions. TMRE staining further corroborated this observation, demonstrating maintained membrane polarization following MSU exposure, whereas cholesterol crystal treatment induced a pronounced loss of TMRE signal. Collectively, the findings highlight a stimulus-specific disruption of mitochondrial dynamics, with cholesterol crystals exerting a distinct and deleterious impact on endothelial mitochondrial homeostasis, potentially amplifying the pathogenic role in vascular inflammation.

Together, these findings establish that crystalline particles elicit multifaceted effects in endothelial cells, including inflammatory activation, altered permeability, and mitochondrial stress. While both CC and MSU are capable of triggering endothelial responses, the nature and extent of these effects appear to be shaped by the concentration and specific cellular pathways engaged. These insights provide a foundation for understanding how crystal accumulation within the vasculature may contribute to inflammation, barrier dysfunction, and disease progression.

A limitation of this study is that it was performed in HUVECs, a widely used model of endothelial biology, although these cells may not fully reflect arterial endothelial responses in atherosclerosis. Complementary studies in arterial endothelial cells and in vivo models will help to further substantiate these findings.

In conclusion, our study demonstrates that cholesterol and monosodium urate crystals induce distinct yet overlapping endothelial responses characterized by inflammatory activation, barrier dysfunction, and mitochondrial stress. These findings highlight endothelial cells as active participants in crystal-driven vascular inflammation and provide a mechanistic basis for understanding how crystalline deposits may contribute to atherosclerotic disease progression. From a translational perspective, these insights suggest that targeting crystal–endothelium interactions or crystal load could represent a novel strategy to mitigate vascular inflammation and reduce early atherogenic risk.

## 4. Materials and Methods

### 4.1. Cell Culture

Human Umbilical Vein Endothelial Cells (HUVECs) were maintained in 75 cm^2^ culture flasks using complete endothelial growth medium (VascuLife basal medium) supplemented with VEGF (LifeFactors Kit; LifeLine Cell Technologies, Carlsbad, CA, USA), along with penicillin (10,000 U/mL) and streptomycin (10,000 µg/mL) (PEST; Life Technologies, Waltham, MA, USA). Cells were incubated at 37 °C in a humidified atmosphere containing 5% CO_2_. The culture medium was refreshed every 48–72 h. Cells between passages 5 and 10 were utilized for all experiments.

### 4.2. CC and MSU Uptake in HUVECs

HUVECs were seeded at a density of 1.5 × 10^5^ cells per well in 12-well tissue culture plates containing complete endothelial growth medium. After 12–16 h of incubation at 37 °C in a humidified 5% CO_2_ atmosphere to ensure cell adherence and stabilization, cells were stimulated with increasing concentrations of cholesterol crystals (CC; 100, 500, 1000, and 2000 μg/mL) or monosodium urate crystals (MSU; 10, 50, 100, and 200 μg/mL). Following 24 h of exposure, culture supernatants were collected and stored at −80 °C for lactate dehydrogenase (LDH) quantification, serving as an index of cytotoxicity. Adherent cells were washed, trypsinized, and resuspended in flow cytometry buffer for downstream processing and viability assessment via flow cytometric analysis.

### 4.3. Stimulation of HUVECs with CC and MSU

HUVECs were seeded at cell densities of 2.5 × 10^5^ cells/well in 6-well plates containing complete endothelial medium. Cells were stimulated with CC (500 µg/mL) or MSU (100 µg/mL) and incubated for either 4 h or 24 h. At the 4 h time point, cells were harvested and immediately snap-frozen at −80 °C for subsequent protein extraction and Western blot analysis. For the 24 h time point, both cell lysates and cell culture supernatants were collected and stored at −80 °C. Total RNA was isolated from harvested cells for quantitative real time PCR (qPCR), while supernatants were utilized for cytokine quantification via ELISA.

### 4.4. Flow Cytometry

HUVECs were exposed to increasing concentrations of CC and MSU for 24 h. Post-treatment, cells were harvested, resuspended in flow cytometry buffer, and centrifuged at 300× *g* for 5 min. Surface staining was performed using fluorescein isothiocyanate (FITC)–conjugated anti-human CD31 monoclonal antibody (BioLegend, San Diego, CA, USA) for 30 min at 4 °C to confirm endothelial identity. Following incubation, cells were washed three times by centrifugation (300× *g*, 5 min) and resuspended in fresh flow buffer. Viability was assessed using 7-Aminoactinomycin D (7-AAD, BioLegend), a DNA-binding dye that selectively penetrates non-viable cells, enabling the exclusion of dead cells during analysis. Crystal internalization was quantified based on changes in side scatter (SSC) properties, indicative of increased intracellular granularity. Gating thresholds were defined using unstimulated controls to determine baseline SSC profiles. The percentage of CD31^+^ cells exhibiting elevated SSC was interpreted as a measure of CC and MSU uptake. Data acquisition was performed using a BD FACSVersé flow cytometer (Becton Dickinson, Franklin Lakes, NJ, USA), and the results were analyzed using Kaluza software, Kaluza version 2.1. (Beckman Coulter, Fullerton, CA, USA).

### 4.5. Lactate Dehydrogenase Cytotoxicity Assay

Cellular cytotoxicity was assessed using the Thermo Scientific™ Pierce™ LDH Cytotoxicity Assay Kit (Thermo Scientific™, Waltham, MA, USA), according to the manufacturer’s protocol. Supernatants from control, CC-, and MSU-treated HUVECs were transferred to 96-well plates in triplicate. An equal volume of reaction mixture was added and incubated for 30 min at room temperature. The reaction was stopped using stop solution, and absorbance was measured at 490 nm and 680 nm. LDH activity was calculated by subtracting background absorbance at 680 nm from 490 nm readings.

### 4.6. Immunofluorescence Staining

To visualize the intracellular localization of CC and MSU in HUVECs, an 8-well chambered glass slide (adhesive silicone mount) was pre-coated with fibronectin (2.13 mg/mL in PBS) for 1 h at 37 °C. Following coating, HUVECs were seeded at a density of 25,000 cells/well in complete endothelial medium and incubated for 24 h at 37 °C in 5% CO_2_. Cells were then treated with CC (500 µg/mL) or MSU (100 µg/mL) for an additional 24 h under identical incubation conditions. After treatment, cells were washed with PBS and fixed with 4% paraformaldehyde for 1 h at room temperature. Permeabilization was performed using 0.025% Triton X-100 in PBS (3 washes), followed by blocking with 1% BSA in PBS-Triton. Cytoskeletal staining was carried out using Rhodamine phalloidin (30 min, RT, dark) and nuclear staining with DAPI (5 min). After air-drying, chambers were mounted with fluorescence-mounting medium and coverslipped. Imaging was performed using a laser-scanning confocal microscope.

### 4.7. Cytokines Measurement Using ELISA

The release of IL-6, IL-8, MCP-1, and CXCL1 in HUVECs were quantified in cell culture supernatant using DuoSet^®^ ELISA kits (R&D Systems, Minneapolis, MN, USA) following the manufacturer’s protocols. Absorbance was recorded at 450 nm using a Cytation 3 microplate reader (BioTek, Winooski, VT, USA).

### 4.8. Western Blot

Western blotting was performed to assess the activation of signaling pathways in HUVECs following CC and MSU stimulation. Total protein was extracted using ice-cold 1× RIPA lysis buffer supplemented with a protease inhibitor cocktail (Thermo Fisher Scientific, Waltham, MA, USA). Protein concentration was quantified using the BCA Protein Assay Kit (Thermo Fisher Scientific) according to the manufacturer’s instructions. Equal amounts of protein (8–10 µg) were denatured in 4× SDS sample buffer at 95 °C for 5 min and separated by SDS-PAGE using 8–16% Criterion™ TGX Stain-Free™ Precast Gels (Bio-Rad, Hercules, CA, USA) with 10× Tris/Glycine/SDS running buffer. Proteins were transferred onto nitrocellulose membranes using the Trans-Blot^®^ Electrophoretic Transfer Cell (Bio-Rad) and 10× Tris/Glycine transfer buffer. Membranes were stained with MemCode™ Reversible Protein Stain (Thermo Fisher Scientific) to verify total protein transfer. After blocking in 5% non-fat dry milk in TBS-T (10 mM Tris-HCl pH 8.0, 150 mM NaCl, 0.1% Tween-20) for 45 min, membranes were incubated overnight at 4 °C with the following primary antibodies: phospho-STAT3 (Tyr705, #9131, 1:1000), phospho-AKT (Ser473, #4060, 1:2000), total STAT3 (#4904, 1:2000), total AKT (#4691, 1:2000), IκBα (#4812, 1:1000) (all from Cell Signaling Technology, Danvers, MA, USA), and GAPDH (FL-335, 1:5000; Santa Cruz Biotechnology, Dallas, TX, USA). After washing, membranes were incubated for 2 h at room temperature with HRP-conjugated anti-rabbit or anti-mouse secondary antibodies. Protein bands were visualized using a Western-Ready™ ECL Substrate Premium Kit (BioLegend, San Diego, CA, USA), and chemiluminescence was detected using the ChemiDoc™ MP Imaging System (Bio-Rad). Densitometric analysis was conducted using Image Lab 5.0 software (Bio-Rad).

### 4.9. Neutrophil Adhesion Assay

#### 4.9.1. Neutrophil Isolation and Labeling

Human neutrophils were isolated from peripheral blood obtained from three healthy donors using density gradient centrifugation with Lymphoprep™ and Polymorphprep™ solutions (Axis-Shield, Oslo, Norway), following the manufacturer’s instructions. Following isolation, neutrophils were enumerated using a hemocytometer and resuspended at the desired concentration. For live-cell tracking, neutrophils were labeled with 1 µg/mL Calcein-AM (Thermo Fisher Scientific) and incubated at 37 °C in a humidified atmosphere containing 5% CO_2_ for 30 min. After staining, cells were washed three times by centrifugation at 300× *g* for 5 min in phosphate-buffered saline (PBS) to remove excess dye. The labeled neutrophils were subsequently resuspended in complete endothelial culture medium and used for adhesion assays. Ethical approval for the isolation of blood from healthy individuals following informed consent was obtained from the Regional Ethics Review Board in Uppsala, Sweden (Dnr 2015/437). Blood collection from healthy donors was carried out in accordance with the ethical principles of the Declaration of Helsinki and the regulations of the Swedish National Board of Health and Welfare.

#### 4.9.2. HUVEC Treatment with Neutrophil

HUVECs were seeded at a density of 2.0 × 10^4^ cells/well in 96-well plates containing complete endothelial growth medium and incubated for 24 h at 37 °C in 5% CO_2_. Cells were then treated for an additional 24 h with conditioned medium, CC, or MSU, according to the experimental design. Following treatment, Calcein-AM labeled neutrophils (1 × 10^5^ cells/well) were added and incubated for 30 min under standard culture conditions. Wells were gently washed twice with flow buffer to remove non-adherent cells. Endothelial growth medium was added to each well, and fluorescence was quantified using a plate reader. Three independent experiments in triplicate were performed for each crystal.

### 4.10. Real Time Quantitative PCR

Total RNA was isolated using the E.Z.N.A.^®^ Total RNA Kit (Omega Bio-Tek, Norcross, GA, USA) in accordance with the manufacturer’s instructions. Complementary DNA (cDNA) was synthesized from 250 ng of total RNA using the High-Capacity cDNA Reverse Transcription Kit (Thermo Fisher Scientific). Quantitative real time PCR (qRT-PCR) was performed to analyze mRNA expression levels of *ICAM1* (assay ID: Hs00164932_m1), *VCAM1* (assay ID: Hs01003372_m1), *SELP* (assay ID: Hs00927900_m1), *SELE* (assay ID: Hs00174057_m1), and *GAPDH* (assay ID:Hs99999905_m1) using TaqMan^®^ Universal PCR Master Mix along with gene-specific TaqMan^®^ primers and probes (Thermo Fisher Scientific, Waltham, MA, USA) on the QuantStudio™ 7 Flex Real-Time PCR System (Thermo Fisher Scientific, Waltham, MA, USA). GAPDH served as the endogenous control for normalization of gene expression levels.

### 4.11. Endothelial Permeability Assay

An in vitro vascular permeability assay kit containing hanging cell culture inserts in a 24-well plate was used (Millipore™; ECM644, Burlington, MA, USA). Cells were cultured and treated according to manufacturer’s instructions. The extent of permeability was determined by measuring the fluorescence (FITC-Dextran; Sigma-Aldrich, St. Louis, MO, USA) of the receiver plate well solution after 1 h using the Gen5 microplate reader.

### 4.12. Detection of Neutrophil Extracellular Traps (NETs) Formation Using Enzyme Linked Immunosorbent Assay

Neutrophils (1 × 10^5^ cells/well) were seeded in 96-well plates and stimulated with CC, MSU, or conditioned medium for 6 h. NET formation was quantified by measuring citrullinated histone H3 levels in the culture supernatant using Citrullinated Histone H3 (Clone 11D3) ELISA Kit, following the manufacturer’s protocol (Cayman chemical, Cat: 501620; Ann Arbor, MI, USA). Three independent experiments in triplicate were performed for each crystal.

### 4.13. Mitochondrial Respiration Using Seahorse

Mitochondrial respiration was evaluated using the Seahorse XF Cell Mito Stress Test Kit (Agilent Technologies, Cheadle, UK) following the manufacturer’s instructions. HUVECs (2 × 10^4^ cells/well), pre-treated with CC or MSU for 24 h, were transferred into Seahorse XF assay medium supplemented with 10 mM glucose, 1 mM pyruvate, and 2 mM L-glutamine to equilibrate prior to mitochondrial stress analysis. Oxygen consumption rate (OCR) was recorded using the Seahorse HA mini-analyzer, with sequential injections of 1.5 μM oligomycin, 2.5 μM FCCP, and 1 μM rotenone/antimycin A to assess basal respiration, ATP production, maximal respiration, and spare respiratory capacity. Three independent experiments in duplicate were performed for each crystal.

### 4.14. TMRE Staining

Mitochondrial membrane potential was assessed using tetramethylrhodamine ethyl ester (TMRE; Thermo Fisher Scientific, CA, USA, cat# T669). Cells were incubated with 50 nM TMRE for 20 min at 37 °C. TMRE selectively accumulates in polarized mitochondria, enabling the assessment of mitochondrial membrane potential as an indicator of mitochondrial integrity.

### 4.15. Statistical Analysis

Graphical data presentation and statistical analyses were performed using GraphPad Prism software (version 9.0, San Diego, CA, USA). Statistical significance was set at *p* < 0.05. Comparisons between two groups were analyzed using unpaired *t*-tests, while multiple group comparisons were conducted using one-way ANOVA followed by Dunnett’s post hoc test for multiple comparisons.

## Data Availability

The data generated in the present study may be requested from the corresponding author.

## Abbreviations

The following abbreviations are used in this manuscript:

CC	Cholesterol crystals
HUVECs	Human umbilical vein endothelial cells
MSU	Monosodium urate crystals
NET	Neutrophil extracellular trap
CM	Conditioned Medium
ATP	Adenosine triphosphate
TMRE	Tetramethylrhodamine ethyl ester
NF-κb	Nuclear factor kappa light chain enhancer of activated B cells
STAT3	Signal transducer and activator of transcription 3
ICAM	Intercellular adhesion molecule
VCAM	Vascular cell adhesion molecule
SELE	Selectin E (E-selectin)
SELP	Selectin P (P-selectin)
NLRP3	NOD-, LRR- and pyrin domain-containing protein 3 (inflammasome sensor)
SMC	Smooth muscle cells
MAPK	Mitogen activated protein kinase
LDH	Lactate dehydrogenase
ELISA	Enzyme linked immunosorbent assay
DAPI	4′,6-Diamidino-2-phenylindole
BSA	Bovine serum albumin
DECT	Dual energy computed tomography
